# Recovery-based staff training intervention within mental health rehabilitation units: a two-stage analysis using realistic evaluation principles and framework approach

**DOI:** 10.1186/s12888-016-0999-y

**Published:** 2016-08-17

**Authors:** Sadiq Bhanbhro, Melanie Gee, Sarah Cook, Louise Marston, Melanie Lean, Helen Killaspy

**Affiliations:** 1 Centre for Health & Social Care Research, Sheffield Hallam University, Montgomery House 32 Collegiate Crescent, Sheffield, S10 2BP UK; 2 Departments of Primary Care and Population Health and Priment Clinical Trials Unit, University College London, London, UK; 3 Division of Psychiatry, University College London, London, UK

## Abstract

**Background:**

Long-term change in recovery-based practice in mental health rehabilitation is a research priority.

**Methods:**

We used a qualitative case study analysis using a blend of traditional ‘framework’ analysis and ‘realist’ approaches to carry out an evaluation of a recovery-focused staff training intervention within three purposively selected mental health rehabilitation units. We maximised the validity of the data by triangulating multiple data sources.

**Results:**

We found that organisational culture and embedding of a change management programme in routine practice were reported as key influences in sustaining change in practice. The qualitative study generated 10 recommendations on how to achieve long-term change in practice including addressing pre-existing organisational issues and synergising concurrent change programmes.

**Conclusions:**

We propose that a recovery-focused staff training intervention requires clear leadership and integration with any existing change management programmes to facilitate sustained improvements in routine practice.

## Background

**Fig. 1:**
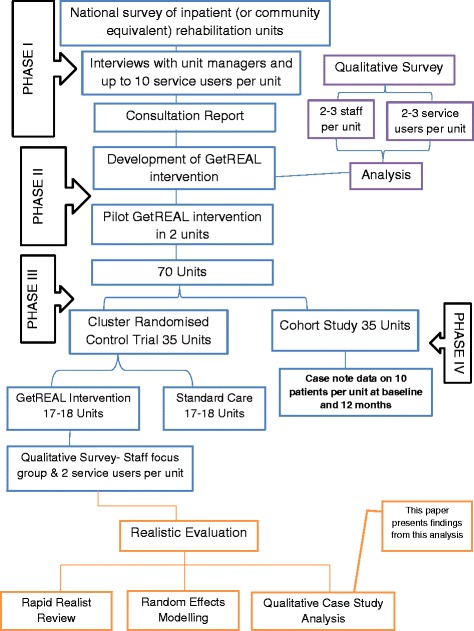
Realistic Evaluation within REAL study plan

In recent years many mental health rehabilitation services have adopted a recovery-based approach, aiming to encompass the values of hope, agency, opportunity and inclusion. This approach values service users as partners in a collaborative relationship with staff who work together to identify and pursue an individual’s personal goals [1]. It also seeks to incorporate service user involvement in service development, staff training and staff appointments [2]. Integral to achieving individuals’ recovery goals is having the opportunity to take part in their chosen activities. As part of a national programme of research into mental health rehabilitation services, the Rehabilitation Effectiveness for Activities for Life (REAL) study, included the development of a training intervention (“GetREAL”) to increase the confidence and skills of staff working in inpatient mental health rehabilitation units in engaging service users in activities (see details in Fig. 1). The intervention has been described in detail elsewhere [3]. In brief, it comprised three stages; predisposing, enabling and reinforcing. In the predisposing stage two senior members of the research team visited each unit to gain local “sign up” and ensure the intervention team would be appropriately supported. The enabling stage involved the intervention team (an occupational therapist, an activity worker and a service user expert) working alongside the rehabilitation unit staff for five weeks to deliver training and modelling in specific processes and skills related to improving service user engagement in activities. At the end of the enabling period an Action Plan was agreed that clarified the changes to structures and processes the unit would continue with and identified a member of staff who would act as the Unit’s “champion”. The reinforcing stage aimed to maintain the new skills the staff had learnt and the changes to structures and process they had agreed on during the enabling stage by providing ongoing, regular email contact between the unit staff and the intervention team for 12 months [3].

The intervention was evaluated through a cluster randomised controlled trial (RCT). Disappointingly, it was not found to be associated with any clinical advantage over usual care and did not increase service user engagement in activities [4]. A qualitative process evaluation that included focus groups with staff at the intervention units revealed that the increased staff skills and changes in practice that were facilitated in units by the GetREAL teams during the enabling stage of the intervention were not sustained during the reinforcing stage (once the GetREAL teams had left the units) [5]. This may explain the lack of effectiveness of the intervention. In a separate component of the REAL programme, over 350 service users were followed for 12 months through a cohort study. Over half were successfully discharged to the community (without readmission or placement breakdown). Factors associated with this included the degree of recovery orientation of the inpatient rehabilitation unit and service user engagement in activities at recruitment [6]. This suggests that the aims and focus of the GetREAL intervention were appropriate and further investigation is therefore justified to understand whether specific aspects of the intervention may require revision to improve its effectiveness.

The evaluation of the GetREAL intervention was designed as a RCT with a view to answering the question ‘does the intervention work?’ As such, fidelity to the intervention was important. We acknowledge, however, that this may not be the most appropriate approach for evaluating complex interventions. Complex interventions attempt to change systems through influencing the behaviour of individuals, and focus on systems that can respond in unpredictable ways, can demonstrate emergence (complex patterns of behaviour arising from relatively simple interactions), and non-linearity of outcomes [7]. Hence, we used a theory-driven evaluation approach that does not rely on a single outcome measure to deliver the verdict on effectiveness of an intervention [8].

There has been growing interest in theory-driven evaluation approaches in health services research. Such approaches emerged during the 1980s within the policy and programme evaluation work by Chen and Rosi [9]. However, they were generally constrained to before-after and input–output designs and were limited methodologically [10]. In recent years, realistic evaluation has appeared as a theory-driven approach with stronger philosophical underpinnings and a focus on theory testing and refinement [11]. The key element of this approach is a programme theory, looking at context-mechanisms-outcomes (CMO) of the programme, that is, what activates mechanisms, amongst whom and in what conditions, to bring about change in the target outcomes [8]. The aim of this approach is to assess not only the effectiveness of an intervention but also the specific elements of an intervention that may contribute to its effectiveness. It asks the questions how or why does an intervention work? ‘for whom does it work?’ and ‘in what circumstances does it work?’ [11].

This paper presents the findings of our qualitative evaluation using a realist approach. It aims to explore the factors associated with variation between units in sustaining the intended recovery-oriented practice during the recovery-focused staff training intervention (GetREAL).

## Methods

We used a qualitative case study analysis using a blend of traditional ‘framework’ analysis and ‘realist’ approach using multiple sources of existing data collected during the REAL programme. We first undertook a rapid realist review of literature (reported separately) to identify candidate programme theories to inform the realistic evaluation.

### Construction of a Sample

A purposive sample of three mental health rehabilitation units was drawn from the 19 units that took part in the cluster RCT and received the GetREAL intervention. The sample of three units was restricted due to time and resource limitations. The objective of the selection of three units was to achieve multiplicity of unit characteristics rather than representativeness. The sample strategy was useful to capture a diversity of perspectives from selected three units. The following purposive selection criteria were used:

### Unit selection criteria

- The unit took part in the cluster randomised controlled trial and received the GetREAL intervention.
- The unit took part in a staff focus group that occurred between 2 and 9 months post intervention (nine of the 19 units that received the GetREAL intervention participated in staff focus groups).
- The unit had either high, mid or low scores in the trial’s primary outcome measure, service user activity as assessed using the Time Use Diary (TUD) at 12 month follow-up [12].
- The unit had a complete dataset containing GetREAL team reflective diaries, staff focus group and service user interview transcripts, unit action plan and fidelity sheet.

Characteristics of the units from which the purposive sample were drawn are shown in Table 1. The table contains characteristics of the 8 units because the unit 9 was closed down before the end of the study.

**Table 1:** Unit characteristics

Unit Code	Difference in Time Use Diary Scores (Follow up minus baseline)	Location	Type	No of beds	Team (staff working on the unit at baseline)		
					Psychiatrist	Psychologist	Occupational Therapist
0102	3	City	Hospital	14	Yes	Yes	Yes
0804	5	City	Hospital	26	Yes	Yes	Yes
**2902**	**−6**	**Suburban**	**Community**	**31**	**No**	**No**	**Yes**
3106	2	Suburban	Community	18	Yes	Yes	Yes
**3301**	**4**	**City**	**Hospital**	**25**	**Yes**	**Yes**	**Yes**
3704	−2	City	Community	20	Yes	Yes	Yes
**4203**	**7**	**City**	**Hospital**	**15**	**Yes**	**Yes**	**Yes**
4204	4	City	Community	15	Yes	Yes	Yes

**Bold**: Selected units for qualitative case study analysis

In consultation with the study statistician (LM) the units 4203 and 2902 were selected as they had the highest and lowest TUD mean scores at 12 month follow-up. The mean TUD score at 12 month follow-up for service users of all eight units was 4. Units 3301 and 4204 both had a mean 12 month follow-up score of 4. Unit 3301 was selected as it was medium size (25 beds) and to represent units that had a median difference in TUD between baseline and follow up.

### Data collection

The data previously gathered during the RCT for the three units selected for the case studies included transcripts of staff focus groups (*n* = 3) and service user interviews (*n* = 4) conducted during the qualitative component of the study; the GetREAL team members’ daily reflective practice diaries (*n* = 26); the unit staff evaluation forms (*n* = 9); fidelity monitoring sheets (*n* = 3) and supervisors notes (*n* = 6) compiled by the GetREAL team members and their supervisors at the end of the enabling stage of the intervention in each unit. Multiple data sources were used in this study in order to aid triangulation. The profiles of selected units were developed based on information drawn from the units’ Action Plans (AP) and intervention fidelity sheets (FS). The stage one framework analysis used reflective diaries (RD), staff focus groups (SFG) and service user interviews (SUI). Theory-led findings were drawn from all data sources and from data collected using the Quality Indicator for Rehabilitative Care (QuIRC), a quality assessment tool used in the RCT to assess services’ performance on seven aspects of care. The QuIRC content was derived from triangulation of findings from three sources in order to identify the components of care that are most important for the recovery of people living in longer term mental health facilities. The final version of the QuIRC is available as a web based application http://www.quirc.eu/ completed by the manager of the unit. The glossary of the data sources is given in Table 2.

**Table 2:** Glossary of data sources

AP	Action Plan
FS	Fidelity Sheet
RD	Reflective Diary
SFG	Staff Focus Group
SUI	Service User Interview
SR	Supervision Record
TEN	Training Evaluation Notes
QUiRC	Quality Indicator for Rehabilitative Care
OTI	Occupational Therapist Instructor
NA	Nursing Assistant
OT	Occupational Therapist
CN	Charge Nurse

### Data analysis

SB and HB carried out the qualitative analysis under supervision of SC. As we were following the ‘realist’ approach to support or challenge the theories identified through the rapid realist review of literature, we used a blend of traditional ‘framework’ analysis and ‘realist’ approaches. The framework approach was used to classify and organise the data according to key themes that emerged from the data [13]. It identified a series of main themes subdivided by a succession of related subthemes and had the benefit of revealing concepts that may not be found in the theories derived from the literature. To focus on theory testing and refinement the next process began with identification of the programme theories to be tested, which were articulated in the form of Context-Mechanisms-Outcome (CMO) configurations. The data were interrogated by the identified candidate theories to see if they could explain the complex footprint of outcomes left by the intervention [11].

Both approaches adopted a realist methodology rather than a phenomenological stance. Within the realist paradigm reality is “real” with a plausible understanding achieved through triangulation from many sources [14]. In a phenomenological paradigm reality is the meaning people give to their lived experiences, which creates a world of multiple constructed realities. Importantly research findings generated using a realist approach can be generalised to theoretical propositions and not to populations [15]. Whereas the findings of phenomenological studies cannot be usefully generalised to other individuals [14].

Qualitative data from the selected three units was managed and stored in NVivo 10 software [16]. Both researchers read the transcripts to familiarise themselves and prepare for the analysis. The initial analysis was carried out using the coding, indexing and charting techniques of the framework analysis approach [13]. This was followed by an iterative process of mapping evidence against theories identified from the literature to challenge or support them. The qualitative case study analysis was carried-out using the following steps:

- All textual data was entered into NVivo v10 software and coded with an index of themes and subthemes.
- The data for each theme was then entered into a matrix to analyse themes across the data sources and cases.
- The contextual profiles of selected units were constructed from the data.
- The identified seven candidate theories from the rapid realist review were tested by plotting evidence against them from available data.
- Final interpretation and synthesis of the emerging patterns and explanations were produced, comparing the 3 cases in relation to the rapid realist review.
- The evidence was interrogated and debated by the three analysts in a series of team discussions.

### Reliability and validity

It is recommended that a detailed and transparent ‘audit trail’ of the processes followed for the evaluation be provided to ensure the reliability of methods and findings [17]. In this study we tried to ensure the validity by data triangulation (that is using different sources of data collected using different methods), and providing an ‘audit trail’ of raw data and steps followed in the analysis including identification of candidate theories. This was reinforced by discussion with team members to verify processes at different stages of the study. However, reliability and validity of each method cannot be guaranteed as all methods have their own potential threats [17]. In this study a potential threat was ‘over-fitting’ of the data due to its scarcity.

## Results

### Unit characteristics

The characteristics of the selected units are shown in Table 3 below.

**Table 3:** Unit profiles

Characteristics	Unit 1 - (Code 4203)	Unit 2 - (Code 3301)	Unit 3 - (Code 2902)
Time Use Diary score at 12 months follow-up	highest	mid-range	lowest
Opened	within last 5 years	more than 15 years ago	the information was not available
Location	suburban hospital	community-based unit in a city	community-based unit in a rural area
No. of beds	15	25	31
Staffing	psychiatrist, clinical psychologist, Occupational Therapist	psychiatrist, clinical psychologist, Occupational Therapist	occupational therapist but no psychiatrist or clinical psychologist
Staff attending GetREAL sign-up meeting (predisposing stage)	unit manager, activity workers, nurses	ward manager, clinical psychologist	unit manager, occupational therapist, activity worker, senior service manager, psychiatrist
Staff attendance at initial GetREAL training workshop	18/24 (75 %)	24/36 (67 %)	28/36 (78 %)
Staff attendance at final GetREAL training workshop	9/24 (36 %)	12/36 (33 %)	8/36 (22 %)

As above table shows that there was a significant difference between “staff attendance in initial GetREAL training workshop” and “staff attendance in final GetREAL training workshop”. We think the reasons behind differences in staff attendance would be: in some units attendance was not made compulsory for staff; in some units executive staff members were present at the ward training to stress the importance of the training; and initial buy-in was not all levels from management to ground-level staff.

### Thematic findings

Table 4 shows the index of initial themes and subthemes used to code the data.

**Table 4:** Themes and Sub-themes Index

Themes	Sub-themes
Predisposing	People involved from units in sign-up
Reception of GetReal	Expectations
	Knowhow prior to training
	Positive views
	General perception of staff about GetReal
GetREAL Training	GetREAL training workshop
	Attendance level
	Staff views on training day
	Staff views on training facilitators/educators
	Fresh perspective
	Staff engagement during training
	Went well during training
	Challenges/issues/gaps
	Improvements for next time
Change in practice	Goal setting
	Planning activities
	Progress in activities
	Meaningful activity
	Motivation for change
	Types of activities
	Links with community teams
Structural changes	Shift patterns
	Changes to structure
Service User engagement in activities	Dealing with challenging people
	Benefits
Dealing with hierarchy	Permission issues
	Barriers
	Managing Continuity
GetREAL legacy	Maintaining the legacy
	Post GetREAL contact
	Success/knock on effect
	Sustainability
	Action plan
	Achieved by GetREAL team

The iterative process of framework analysis generated the following four main themes that appeared to contribute to either the maintenance or inhibition of long-term change.

#### Readiness for the intervention

Lack of clarity about the purpose and content of the intervention and fear of being scrutinised caused some staff in the units to feel apprehensive and confused about the GetREAL teams’ arrival. This may have impeded the process of embedding changes in practice.

There was some inconsistency across units in terms of how well informed staff were about the GetREAL intervention before the GetREAL team arrived on the Unit. Some staff, mainly those who had attended the sign-up meeting, had heard that the GetREAL team was coming and were clear about the purpose of the intervention but some were unsure about the purpose and practical implications. For instance,

*Many of the staff didn’t know much about the REAL programme* (RD GetREAL OT: Unit 1).

*We weren’t aware of what it* [GetREAL] *was about …it was do it with activities and their* [patients] *mental health and stuff, but, actually sort of what people are going to, what the intervention was* (SFG Deputy Manager: Unit 2).

*We just had a few days’ notice that they* [GetREAL team] *were coming, but we didn’t actually know what they were about* (SFG Health Care Assistant 4: Unit 3).

Conversely, the ward manager of unit 1 had positive expectations from the start as the GetREAL OT interpreted that:

*It was a good thing to have intensive involvement for a few weeks from REAL and thought this was valuable and helpfully was keen to embed with staff members who are closely involved* (RD GetREAL OT: Unit 1).

However, in the same unit, the occupational therapist said during a staff focus group.

*I was very apprehensive about them* [GetREAL team] *coming and thinking, oh god, someone is going to be coming watching over us, rather than giving or working with us and judging what I’m doing?* (SFG OT: Unit 1).

#### Maintaining initial enthusiasm

Despite the mixed views about how information about the intervention was shared prior to the GetREAL team’s arrival, once there, staff were generally positive about the intervention team. Staff at all three units appreciated the stimulating effect of ‘outsiders’ in making them review their practice. They also enjoyed seeing service users responding positively to the changes and reported greater confidence in their approach to engaging service users in activities (SFG: Unit 1, 2 & 3). For example, unit 3 staff participated in the focus group remembered how the GetREAL training team made them very enthusiastic to start different activities with service users (e.g. dancing, attending the local gym).

Staff also reported that they had enjoyed the training sessions delivered by the GetREAL team and felt listened to and supported by the team in thinking though how to enact change. Furthermore, they reported that the changes they made during the enabling stage were sustained and further developed over the next few months. Conversely, service user interviews revealed how resource issues had led to activities being stopped (SUI Service User: Unit 3).

This point corroborated findings from our rapid realist review, where we identified that one of the mechanisms for lasting change was staff members feeling ‘resourced for recovery’ (Melanie Gee, personal communication, November 16, 2015).

#### Impact of GetREAL

There were several positive impacts that GetREAL had on the units by the end of the five-week enabling stage.

Focus group participants from all three units reported that after the GetREAL intervention, staff felt energised and motivated; more confident and empowered, and that they knew their patients better (SFG: Unit 1, 2 & 3). Other positive impacts included more collaborative working, improved staff skills and being able to offer a wider variety of activities to service users.

*GetREAL has promoted staff and service users’ involvement in activities, which is valuable* (SFG Charge Nurse: Unit 1).

*After GetREAL the unit staff have a better understanding of the complexities of the unit* (SFG GetREAL OT: Unit 2)

*It [GetREAL] had an enormous benefit in that it was joint working and collaborative working and that it was going to bring everyone together, as one team, working in one direction, and offering the service users here a greater range of meaningful activities, not necessarily just groups but meaningful activity, in the widest sense* (SFG OT instructor: Unit 3).

Service users also appeared to benefit and to gain confidence to ‘speak up’. They also gave positive feedback about the increased focus on activities:

#### The GetREAL Legacy

The evidence for sustained change in practice as a result of the GetREAL intervention was mixed. Facilitating factors appeared to be involvement of all staff; positive feedback from service users and regular review of the Action Plan. Barriers included lack of staff to support a range of activities and staff being too busy to extend their job roles to include facilitating activities.

On unit 1, some of the activities initiated during the enabling stage that had involved all staff were continued, such as a gardening group at a local allotment. It appears that sustaining this activity was helped by the positive feedback from service users about how much they enjoyed it. Also on unit 1, staff continued to review their Action Plan regularly. However, in unit 3 staff focus group participants noted that sometimes there were not enough staff to facilitate groups on the unit (SFG OT instructor: Unit 3).

In unit 2, staff admitted that ‘*nobody actually took over where the GetREAL team left off because everyone has got enough on their job roles’* (SFG Deputy Manager: Unit 2).

These themes were then mapped onto the ‘Context Mechanism Outcome’ (CMO) configuration derived from our rapid realist review (see Table 5).

**Table 5:** Initial themes from the qualitative analysis of the case study data

Theme	Context	Mechanism	Outcome
Reception of GetREAL	Lack of prior information and engagement that involved all staff	Delay in staff engaging with the short term intervention	Only short term changes
Maintaining initial enthusiasm	Looking afresh and starting new activities	Stimulating strong enthusiasm and seeing service users respond positively	Carrying on new activities long term
	The training was interesting and collaborative	Felt engaged, listened to and supported	Short term changes
	Lack of equipment and staff time	Service user disappointed because they were not able to continue activities they liked.	New activities stopped
Impact of GetREAL	GetREAL featured: Predisposing meeting to engage managers and senior staff; Enabling stage with trainers working alongside each unit team for 5 weeks to deliver a tailored programme	Staff felt energised and motivated; more confident and empowering, and that they knew patients better.	More collaborative working, improved staff skills in the short term.
	GetREAL featured: Modelling ways to involve service users in developing the service	Service users started having a voice more, and giving positive feedback on the increased activities, which pleased staff.	Wider variety of activities offered to service users and their involvement was encouraged in the short term
The Legacy of GetREAL	Involvement of all staff	Staff engagement in activities was set as a norm.	The evidence for a long-term legacy following the GetREAL training was mixed. Some new activities continued long term
	Positive feedback from service users	Services users enjoyed the activities and were happy to keep them continue.	
	Regular review of the action plan.	Joint planning and working	
	Lack of staff available to support a range of activities and staff being too busy to extend their job roles.	No role flexibility	

### Theory-led findings

**Fig. 2:**
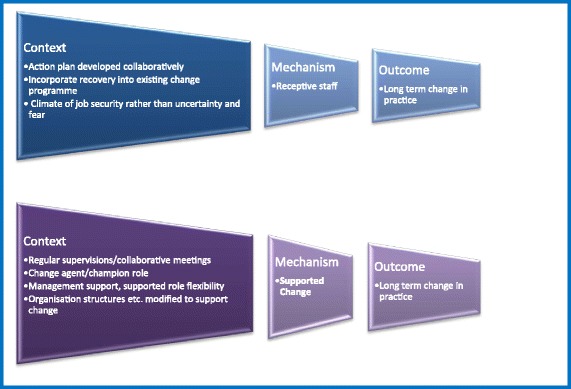
Priority theories from the literature used to interrogate the case study data

In the rapid realist review, we prioritised seven programme theories (CMO configurations). These are shown in Fig. 2. We present below a statement of each priority theory and describe to what extent the available data supported or refuted the theory, providing illustrative quotes.

## Receptive Staff (RS)

### Action plan developed collaboratively

Collaborative action-planning between staff groups and service users (C) (in particular where the action plan utilises existing strengths of the individuals concerned (C) leads to staff feeling engaged, valued, and involved (M), and hence ‘Receptive to Change’ (M). Imposing an action plan on staff members (C) will block staff ‘receptiveness’ (M).

The data clearly show that Action Plans in the selected three units were developed collaboratively with all staff members and included management and service users (FS Unit 1, 2 & 3). Furthermore, the unit with the highest outcome scores continued using their Action Plan long term.

Staff found collaborative action planning useful and considered it helpful in considering their future strategy (SFG, AP & FS Unit 1, 2 & 3). In terms of the long-term change the focus group participants of unit 1 (highest long term outcome scores) confirmed that that staff members of the unit were still reviewing their action plan by adding more things and progress updates after 6 months of the GetREAL (SFG: Unit 1). This infers that reviewing over the long term was built into their structures. However, in case of unit 2 and 3 staff (mid-range and lowest long term outcome scores) focus group participants confirmed that the staff members on these units were not using/updating the action plan after GetREAL (SFG: Unit 1 & 2).

#### Illustrative Quotes

*In team meetings staff were encouraged to think and give ideas how patients can be more involved in activities and those can be included in the action plan* (RD GetREAL OT: Unit 1).

*The staff appreciated the action plan review and its focus on success of the team and individuals. They also particularly responded to the way it communicated what had been done to everyone* (RD GetREAL OT: Unit 2).

*Service users (SUs) were responding positively to use and sharing information that is new to the team- have commented on the action plan and were seeing the value of different approaches in action plan* (RD GetREAL OT: Unit 3).

### Incorporate recovery into existing change programme

Incorporating recovery into an existing change programme (C) may help with staff engagement, enthusiasm, and change ‘receptiveness’ (M), in an organisation subject to much recent change (C).

All three units had existing ‘change programmes’ in place before the GetREAL intervention began. In all three units, recovery-based training had been implemented for staff. In Unit 1, the GetREAL training was integrated with an existing change programme (‘Productive Ward’). This helped to embed both sets of changes into longer-term practice. It was also felt to facilitate increased engagement of service users in activities, improve communication between staff when planning activities for individual patients as well as freeing up staff time for care and enabling nursing assistants (NAs) to do more activities (SFG Charge Nurse: Unit 1). Here, the staff maintained their enthusiasm for both change programmes, increased their participation in formal recovery-based training and scored higher on the final 12 month outcome measure assessing the impact of the GetREAL intervention than the other two units. It was recorded that the percentage of staff attending recovery-based training increased from 19 to 85 % over the 12 months of the GetREAL intervention. Unit 2 had implemented a ‘Recovery Model’ prior to the GetREAL intervention but this was felt to have had no impact on practice (RD GetREAL OT: Unit 2), despite the fact that almost all staff had attended this training by the end of the 12 month GetREAL intervention period. Unit 3 had also implemented the ‘Productive Ward’ programme prior to starting the GetREAL intervention and although staff felt that the latter complemented the former through its focus on activities (SFG Staff Nurse: Unit 3), uptake of recovery-based training remained very low (fewer than 10 % of staff attended during the 12 month period).

#### Illustrative Quotes

*Engagement in activities was increased with the start of GetREAL because the productive ward already made the ward environment better* (SFG Charge Nurse: Unit 1).

*The existing recovery model had no impact on practice* (RD *GetREAL OT: Unit 2*)*.*

*The GetREAL programme was building on those things they were already done with the productive ward* (SFG Staff Nurse: Unit 3).

### Climate of job security rather than uncertainty and fear

Overwhelming negative external contextual factors (e.g. economic cutbacks and job uncertainty) (C) will prevent staff members feeling involved, engaged, or valued (M) and hence block their ‘receptiveness’ (M) to a change programme.

The data analysis found insufficient and incomparable evidence to either support or challenge this theory. On Unit 2, two key staff members were on long term sick leave and this had affected the overall ward environment and impeded the staff from involving service users in activities (RD GetREAL OT: Unit 2). This may have impacted on the morale of remaining staff, or reflect the impact of a climate of job insecurity, but insufficient information was available to clarify this. No relevant evidence was found from Unit 1 and Unit 3 data on this issue.

#### Illustrative Quote

*Long term sickness of the activity worker made some staff feel that they have to burden themselves with organising activities on top of their regular duties. The long term off sick of the ward manager had a negative impact on how well everything is organised and whether everything runs according to plan* (RD GetREAL OT: Unit 2).

## Supported Change (SC)

### Regular supervision

Regular meetings between staff groups and the training team, and/or a local change lead (‘champion’) (C), within a supportive organisational culture (C), help staff members feel supported by their peers and managers in the change programme (M).

Some aspects of this theory are supported by the data. In all three units, individual and group supervision was set as the norm and was regularly maintained and appeared to contribute to a supportive organisation. However, we did not find information about how useful the supervision meetings were and whether staff members felt supported or not in implementing changes to practice.

On all three units all clinical staff members had a named supervisor. On Unit 1 staff were recorded as having one-to-one supervision meetings at least weekly at baseline and every 2 to 6 weeks at 12 months follow-up. Staff members and supervisors had group supervision meetings every 2 to 6 weeks (QUiRC). On Unit 2 they had one-to-one supervision meetings every 2 to 6 weeks at baseline and this was maintained at 12-months follow-up. It was reported that at baseline group supervision was not used, but by the 12-months follow-up, group supervision was held every 2 to 3 months (QUiRC). On Unit 3 they had one-to-one supervision meetings every 2 to 6 weeks both at baseline and follow-up. Group supervision was frequently used and they had meetings weekly or more often at baseline and 12-months follow-up (QUiRC). Data were only available from the QUiRC responses as completed by managers of the units.

### Appointing a change agent or ‘champion’

A local change agent or ‘champion’ (C), if supported by management in that role (C) may help to persuade, encourage, and empower (M) other staff members to change - i.e. they feel ‘supported’ to change (M). To be effective, a champion will need to have programmatic optimism, good interpersonal skills, the respect of colleagues, and be influential (C).

This theory was partially supported by the available data, in that although the idea of a champion existed in all three units, the lack of specific, trained and supported change agent/champion posts was associated with poorer long term outcomes in unit 3. As mentioned earlier, built into the GetREAL intervention was provision for a nominated person in each unit to make email contact with the GetREAL training teams in the 12 month follow-up period after they left the unit. However, this role was new to those volunteering for it and they had no formal training or face-to-face support from the GetREAL teams to support them. In Unit 1 it was mentioned that a nursing assistant was identified as the “champion” (RD GetREAL OT: Unit 1). In Unit 2 an enthusiastic OT and psychologist who understood the aims of the GetREAL intervention were considered ‘champions’ but not appointed formally (RD GetREAL OT: Unit 2). On Unit 3 no staff member appeared to have actually been identified as the ‘champion’ (FGD OT: Unit 3).

#### Illustrative Quotes

*A nursing assistant has been identified as a champion for the cause and is doing some good work* (RD GetREAL OT: Unit 1).

*The full-time psychologist and an OT are enthusiastic; rehabilitation focused and really gets the GetREAL concepts* (RD GetREAL OT: Unit 2).

*No one from staff was identified as a champion* (SFG Health Care Assistant: Unit3).

### Management support, and role flexibility

Explicit management endorsement and prioritisation of the change (e.g. through getting involved in the programme; endorsing an action plan for change; measuring progress; incorporating external drivers for change) (C) helps staff members feel supported to make the change (M) even if it entails moving outside their traditional occupational role and taking some risks (C).

In relation to this theory some support was found from the data, in that the management teams on Units 1 and 2 actively endorsed GetREAL as a change programme, whilst on Unit 3 this was lacking.

A predisposing (‘sign-up’) meeting was held with each unit’s manager and senior staff team members to encourage them to support the GetREAL intervention. Dates of staff training days and arrangements to release staff to attend these were agreed in advance by the senior staff (FS Unit 1, 2 & 3). In all three units managers were also involved in the development and endorsement of Action Plans.

On Unit 1 the senior management and nurse manager were actively promoting the intervention from the start despite some of the multi-disciplinary team questioning what could be achieved by their patients. On Unit 2 the manager actively supported the programme, promoted role sharing and proactively involved service users in planning activities. On Unit 3, though the management team and OT attended the predisposing (sign-up) meeting, the nurses and OT later said that they did not know what to expect and what was expected of them (SFG OT: Unit 3). On Unit 3, in contrast to Units 1 and 2, the manager did not mandate that all staff should attend the training sessions (RD GetREAL OT: Unit 3). This may have implied that the manager did not fully support the programme or give it adequate priority d. Consequently; relatively few staff attended the final training.

#### Illustrative Quotes

*The senior management were truly multidisciplinary team in their approaches and despite the pervasive medical model the medical staff members were very involved in the GetREAL* (RD GetREAL OT: Unit 1).

*The leadership team have decided to include key actions from the REAL study in one inclusive document bringing together a range of strategic pieces of work* (RD GetREAL OT: Unit 1).

*Reception in the leadership meeting was positive and supportive. The acting manager showed her support for the team increasingly being involved in role sharing around activities… was very proactive in engaging service users in activity planning discussions* (RD GetREAL OT: Unit 2).

*Most senior staff referred positively to the intervention and said it was useful to get everyone re-think about activities or get some support regarding this* (RD GetREAL OT: Unit 2)

*The manager decided that staff should be invited but not directed to attend. She wanted to see the buy in & promote ownership of change, but for the GetREAL team it did not give the opportunity to work with the more reluctant members and engage in team problem solving* (RD GetREAL OT: Unit 3).

### Modify organisational structures to support change

If organisational structures, processes and systems (e.g. working practices, responsibilities, policies, documentation, and performance reviews) are modified (C) to facilitate the move towards recovery-based practice, staff members will feel supported by management (M) in changing their practices.

There was some evidence that Unit 1 appeared to have more facilitative structures to support the intended changes than the other two units. However, sufficient data were not available to test this theory.

Unit 1 had existing structures that facilitated multidisciplinary planning. They could also make modifications to their systems flexibly in order to enhance the staff and patient experience. The GetREAL team observed that at the time of GetREAL intervention, Unit 2 had structural and management issues such as inadequate/infrequent staff supervision and a lack of line management and performance management in place (RD GetREAL OT: Unit 2). Unit 3 was a large unit with quite a hierarchical structure and rigid staff roles. For instance, nursing assistants felt they had to get permission to undertake fairly simple tasks and they did not feel it was their job to facilitate activities (SFG Health Care Assistant: Unit 3).

#### Illustrative Quotes

*The unit has a well-functioning multidisciplinary team* (*MDT*) *that plans together at fortnightly CTMs* (RD GetREAL OT: Unit 1).

*The unit staff members have already begun making changes to some of their systems and show a willingness and motivation to make things work even better for them and the patients* (RD GetREAL OT: Unit 1).

*The staff make constant reference to the problems with communication in the team. The deputy ward manager is procedure focused and has clearly not stepped in to the supervisory role left by the manager being off sick. This leaves a gap in support/encouragement for the staff to take ownership of their practice development* (RD GetREAL OT: Unit 2).

*I think there were some questions perhaps about what meaningful activity was for people, what counted and certainly looking at the outcome of the first report and matching that against national standards and seeing where we came there, I thought “oh that really doesn’t feel like what goes on at all…* (RD Staff Nurse: Unit 3).

## Discussion

This analysis concluded that there was clear support in the data for two theories that may contribute to long term change in recovery oriented practice: having action plans that are developed collaboratively with all staff and service users and reviewed continuously; and incorporating new change programmes into any existing change programmes. Four theories were partially supported by the data: having regular staff supervision; having a designated ‘change champion’; having managerial support for role flexibility; and having the possibility of modifying organisational structures to support change. We found little evidence to support or refute the theory that a climate of job security rather than uncertainty and fear impacted on long term change.

Our study has several limitations that need to be taken into account when interpreting our findings. First, we purposively sampled three services on the basis of a range of characteristics that we felt may be relevant to our study. These services were not representative of all mental health rehabilitation units across England and our findings may therefore reflect the characteristics of this particular small sample of units. Second, we drew on existing data sources generated through the REAL study and were thus limited to some degree by this in terms of how well these data could help us in our aim of understanding whether the GetREAL intervention had scope for refinement to strengthen its effectiveness.

Further, literature suggests that it is essential to design training programmes which are well aligned with “conceptual dimensions of recovery” [18, 19], and organisations should be careful about relying on staff training programmes which are unlikely to be adequate to create pervasive and long-term change per se [20].

A synergistic view emerged from both stages of the analysis, which suggested that engagement of all staff members (staff “on the ground” as well as management) from the very start of the intervention is needed to ensure acceptance and ownership of change in practice. This process was thwarted by the need for the researchers to remain blinded to whether the unit had been allocated to receive the GetREAL intervention, such that unit staff could not be told about the intervention until after baseline data had been gathered. Gilburt and colleagues (2013) in their quasi-experimental mixed-method study on promoting recovery-oriented practice in mental health services found that front line staffs are the primary change agents in implementing recovery-oriented practice [20]. This process of informing and engaging staff of all levels about the purpose and process of the GetREAL intervention at an early stage of implementation would be a relatively straight forward refinement.

Our analysis found that creating opportunities for staff members to reflect together, obtain feedback, monitor their progress and identify areas for further change helped them feel that their work was a shared responsibility. The involvement of current and former service users in the design and delivery of the intervention was also a powerful illustration that recovery and collaboration is achievable and realistic for service users. In addition, staff engagement in implementing change needs to be supported by adequate resources.

Analysis of the Unit 1 data revealed features associated with an organisational culture that was helpful in sustaining change; staff members were on board from the start of GetREAL, they jointly developed and reviewed action plans and embedded GetREAL with an existing change programme. Supervision and collaborative meetings happened routinely and staff continued updating and using their action plans in the longer term. Both stages of analysis explicitly identify these kinds of practices, as key in creating an organisational culture that could sustain longer-term change in practice.

Findings from both stages emphasised that value was placed on a collaborative goal working approach and service user feedback, as this improved inter-staff relationships, performance and a sense of shared ownership. Similarly, literature suggests that collaborative goal setting and working within recovery-oriented practice is an effective way of gaining service user ownership of the recovery process [21]. Management support featured strongly throughout both stages of analysis, which inferred that levels of management support for a change programme might impact on its long-term sustainability. This was specifically regarding organisational structures, staff role flexibility, and embedding the role of a champion or change agent in permanent posts rather than individual staff members who may leave. Brian and colleagues [22] suggest that occupational therapists potentially can take this new role of change agents to drive recovery-oriented practice in a multidisciplinary team by utilising their core professional values and competencies [22].

The findings of our framework approach and theory testing demonstrated that in the context of long-term change, there was no single measure that sustains long-term change in practice for NHS rehabilitation units. Rather, that several interconnected measures need to be considered prior, during and after a new programme is introduced.

It may be conjectured that in some organisational settings there may be overwhelming problems that would need to be remedied before a training/change intervention would be worth undertaking. Therefore, in addition to tailoring the GetREAL intervention to the individual units, and including realistic evaluation in the methodology, we propose that it would be useful to do some initial, pre-intervention exploration of the organisation. This would serve to identify any organisational, structural or staff team issues that might present fault lines when the team is placed under the additional strain of the intervention. A menu of options could then be provided including a pre-GetREAL programme of change targeted at organisational and structural problems.

### Strengths and limitations of the methods, and future research directions

The choice of realist methodology to evaluate the GetREAL intervention has been vindicated through a demonstration of the complexity of the system. The use of a rapid realist review to generate candidate programme theories proposing the relationships between context and mechanism leading to long-term change has been instrumental to the evaluation process, particularly when dealing with a scarcity of programme data to evaluate. Without the rapid realist review to generate the candidate programme theories, there would have been a danger of ‘over-fitting’ the data [23] and our findings would have limited generalizability even to other units within the study. We used wide sources of data including staff focus groups, service user interviews, fact sheets, reflective diaries etc., however a major limitation of the study was that this available qualitative data was not fit for the purpose as the data was not collected with realist evaluation in mind, to perform a realist evaluation. As such, the data available (as exemplified by the illustrative quotes) did not neatly fall into configurations of Context, Mechanism, and Outcome (CMOs). Further, the rapid realist review of literature was conducted to draw the CMOs for the intervention, the data presented here to test the candidate theories are not considered as causal mechanism because they were not extracted from the data transcripts. A realist evaluation of such an intervention would have involved data collection with the candidate programme theories under scrutiny in mind: focus groups and interviews, with appropriate questions being posed, could be used to explore and refine these theories [24]. Another limitation of the study was that all qualitative data analysis is subject to the individual perspective/s of the researcher on the allocation of text to codes. We tried to minimise this by having two sets of coders etc.

### Recommendations

Whilst acknowledging the complexity of the interactions between contexts and mechanisms, and that data (from the literature and from the GetREAL intervention) was constrained, we suggest the refinements to the GetREAL intervention:

1. Pre-intervention exploration to identify potential problems and the option of offering preliminary organisational change strategies.
2. Initial buy-in for all disciplines, at all levels (management to people on ground).
3. Attendance at training workshops is mandatory to show managers are prioritising it and to engage reluctant staff.
4. Structures in place for maintaining service user involvement in the planning and delivery of their service. This may for example, include SU group meetings and posts for service user development workers.
5. There needs to be sufficient staff time to engage in activities with SUs, for example through flexibility of working patterns.
6. Staff need to record the amount of service user activity they are engaged in, both as a way for staff and service users to feel rewarded and acknowledged, and in a way that is meaningful for commissioners.
7. The long-term role of change agent or champion needs to be clearly designated as part of the team, rather than this function being associated with an individual staff member (who may leave).
8. Ensure any other existing change programmes (e.g. the Productive Ward programme) can embed a complex intervention (e.g. GetREAL) in a combined long-term change process.
9. The Occupational Therapist needs to have the skills and support to engage the multidisciplinary team in activities as part of everyone’s role.
10. Sufficient staff are required and a creative flexible approach to using staff time.

## Conclusions

The realistic evaluation has offered useful directions for long term change programmes by proposing that a recovery-focused staff change intervention requires pre-intervention exploration of organisational culture; tailoring the intervention to specific settings; integration with any existing change programme; and embedding the intervention into routine practice for sustainability. The realistic evaluation must be included in the methodology from the start.

## References

[1] Joint Commissioning Panel for Mental Health (2012). Guidance for commissioners of rehabilitation services for people with complex mental health needs.

[2] Wolfson P, Holloway F, Killaspy H (2009). Enabling recovery for people with complex mental health needs: a template for rehabilitation services in England.

[3] Cook S, Mundy T, Killaspy K (2015). Development of a staff training intervention for inpatient mental health rehabilitation units to increase service users’ engagement in activities. Br J Occup Ther.

[4] Killaspy H, Marston L, Green N (2015). Clinical effectiveness of a staff training intervention in mental health inpatient rehabilitation units designed to increase patients’ engagement in activities (the Rehabilitation Effectiveness for Activities for Life [REAL] study): single-blind, cluster-randomised controlled trial. Lancet Psychiatry.

[5] Lean M, Leavey G, Killaspy H (2015). Barriers to the sustainability of an intervention designed to improve patient engagement within NHS mental health rehabilitation units: a qualitative study nested within a randomised controlled trial. BMC Psychiatry.

[6] Killaspy H, Marston L, Green N (2016). Clinical outcomes and costs for people with complex psychosis; a naturalistic prospective cohort study of mental health rehabilitation service users in England. BMC Psych.

[7] Moore G, Audrey S, Barker M, et al. Process evaluation of complex interventions. UK Medical Research Council (MRC) Guidance; 2014. https://www.mrc.ac.uk/documents/pdf/mrc-phsrn-process-evaluation-guidance-final/. Accessed 22 Dec 2015. 10.1136/bmj.h1258 PMC4366184 25791983

[8] Pawson R, Tilley N (1997). Realistic Evaluation.

[9] Chen H, Rossi P (1983). Evaluating with sense: the theory-driven approach. Evaluation Rev.

[10] Health Care Management Unit http://www.itg.be/internet/ds/tde/tde.html. Accessed 13 Mar 2016.

[11] Pawson R, Tilley N (2004). Realist Evaluation.

[12] Jolley S, Garety PA, Ellett L (2006). A validation of a new measure of activity in psychosis. Schizophr Res.

[13] Ritchie J, Lewis J (2003). Qualitative research practice: a guide for social science students and researchers.

[14] Sobh R, Perry C (2006). Research design and data analysis in realism research. Eur J Marketing.

[15] Yin RK (1994). Case Study Research - Design and Methods.

[16] QSR International Pty Ltd. NVivo qualitative analysis software. 2012;10.

[17] Robson C (2002). Real World Research.

[18] Leamy M, Bird V, Boutillier CL, Williams J, Slade M (2012). Conceptual framework for personal recovery in mental health: systematic review and narrative synthesis. Br J Psychiatry.

[19] Whitley R, Drake RE (2010). Recovery: A dimensional approach. Psychiatric Servicers.

[20] Gilburt H, Slade M, Bird V, Oduola S, Craig TKJ (2013). Promoting recovery-oriented practice in mental health services: a quasi-experimental mixed-methods study. BMC Psychiatry.

[21] Oades L, Deane F, Crowe T, Lambert GW, Kavanagh D, Lloyd C (2005). Collaborative recovery: an integrative model for working with individuals who experience chronic and recurring mental illness. Australas Psychiatry.

[22] Brian H, Cook S, Taylor D, Freeman L, Mundy T, Killaspy H. Occupational therapists as change agents in multidisciplinary teams. Br J Occup Ther. 2015:1–9. doi:10.1177/0308022615586785.

[23] Hawkins A (2014). The case for experimental design in realist evaluation. Learning Communities. Int J Learning in Soc Contexts.

[24] Manzano A (2016). The craft of interviewing in realist evaluation. Evaluation.

